# Efficacy and safety profile of mucolytic/antioxidant agents in chronic obstructive pulmonary disease: a comparative analysis across erdosteine, carbocysteine, and N-acetylcysteine

**DOI:** 10.1186/s12931-019-1078-y

**Published:** 2019-05-27

**Authors:** Paola Rogliani, Maria Gabriella Matera, Clive Page, Ermanno Puxeddu, Mario Cazzola, Luigino Calzetta

**Affiliations:** 1 Unit of Respiratory Medicine, Department of Experimental Medicine, University of Rome “Tor Vergata”, Via Montpellier 1, 00133 Rome, Italy; 2 Unit of Pharmacology, Department of Experimental Medicine, University of Campania “Luigi Vanvitelli”, Naples, Italy; 3 Sackler Institute of Pulmonary Pharmacology, Institute of Pharmaceutical Science, King’s College London, London, UK

**Keywords:** COPD, Erdosteine, Carbocysteine, N-acetylcysteine, Meta-analysis

## Abstract

**Background:**

To date there are no head-to-head studies comparing different mucolytic/antioxidant agents. Considering the inconsistent evidence resulting from the pivotal studies on mucolytic/antioxidant agents tested in chronic obstructive pulmonary disease (COPD), and the recent publication of Reducing Exacerbations and Symptoms by Treatment with ORal Erdosteine in COPD (RESTORE) study, we have performed a meta-analysis to compare the efficacy and safety of erdosteine 600 mg/day, carbocysteine 1500 mg/day, and N-acetylcysteine (NAC) 1200 mg/day in COPD.

**Methods:**

A pairwise and network meta-analyses were performed to assess the efficacy of erdosteine, carbocysteine, and NAC on acute exacerbation of COPD (AECOPD), duration of AECOPD, and hospitalization. The frequency of adverse events (AEs) was also investigated.

**Results:**

Data obtained from 2753 COPD patients were extracted from 7 RCTs published between 2004 and 2017. In the pairwise meta-analysis mucolytic/antioxidant agents significantly reduced the risk of AECOPD (RR 0.74 95%CI 0.68–0.80). The network meta-analysis provided the following rank of effectiveness: erdosteine>carbocysteine>NAC. Only erdosteine reduced the risk of experiencing at least one AECOPD (*P* < 0.01) and the risk of hospitalization due to AECOPD (*P* < 0.05). Erdosteine and NAC both significantly reduced the duration of AECOPD (P < 0.01). The AEs induced by erdosteine, carbocysteine, and NAC were mild in severity and generally well tolerated. The quality of evidence of this quantitative synthesis is moderate.

**Conclusions:**

The overall efficacy/safety profile of erdosteine is superior to that of both carbocysteine and NAC. Future head-to-head studies performed on the same COPD populations are needed to definitely confirm the results of this meta-analysis.

**Trial registration:**

CRD42016053762.

## Background

The regular treatment with mucolytic/antioxidant agents such as erdosteine, carbocysteine, and N-acetylcysteine (NAC) is recommended in patients with chronic obstructive pulmonary disease (COPD) [1]. In fact, the last Global Initiative for Chronic Obstructive Lung Disease (GOLD, 2019) document indicates that mucolytic/antioxidant drugs may reduce the risk of acute exacerbation of COPD (AECOP) and improve health status [1].

Until 2015 there was no evidence to precisely identify the target population for mucolytic/antioxidant agents in COPD [2, 3], and a recent not pre-specified post-hoc analysis PANTHEON study (Placebo-controlled study on efficAcy and safety of N-acetylcysTeine High dose in Exacerbations of chronic Obstructive pulmoNary disease) provided conflicting evidence concerning the role of inhaled corticosteroid (ICS) therapy and smoking habit on the protective effect of high-dose NAC against the risk of AECOPD [4]. Conversely, a quantitative synthesis of randomized controlled trials (RCTs) performed in agreement with the Preferred Reporting Items for Systematic Reviews and Meta-Analyses (PRISMA) Statement demonstrated, via a meta-regression analysis, that the only factors that could significantly influence the efficacy of mucolytic/antioxidant agents in COPD were the quality of RCTs, the duration of treatment, and the number of AECOPD in the year previous the study enrolment [5]. Interestingly, the recent Reducing Exacerbations and Symptoms by Treatment with ORal Erdosteine in COPD (RESTORE) study [6] showed that erdosteine was effective in reducing the rate and duration of AECOPD irrespective of event severity and concomitant ICS treatment.

To date no head-to-head RCTs across different mucolytic/antioxidant agents have been conducted to directly compare the efficacy profile of erdosteine, carbocysteine, and NAC. Therefore, in the light of the inconsistent evidence resulting from the pivotal RCTs on mucolytic/antioxidant agents tested in COPD [7–10], and the recent publication of RESTORE study [6], we have performed a pairwise and network meta-analysis of the currently available data aimed to compare the real efficacy of erdosteine, carbocysteine, and high-dose NAC on AECOPD.

## Materials and methods

### Search strategy

**Fig. 1:**
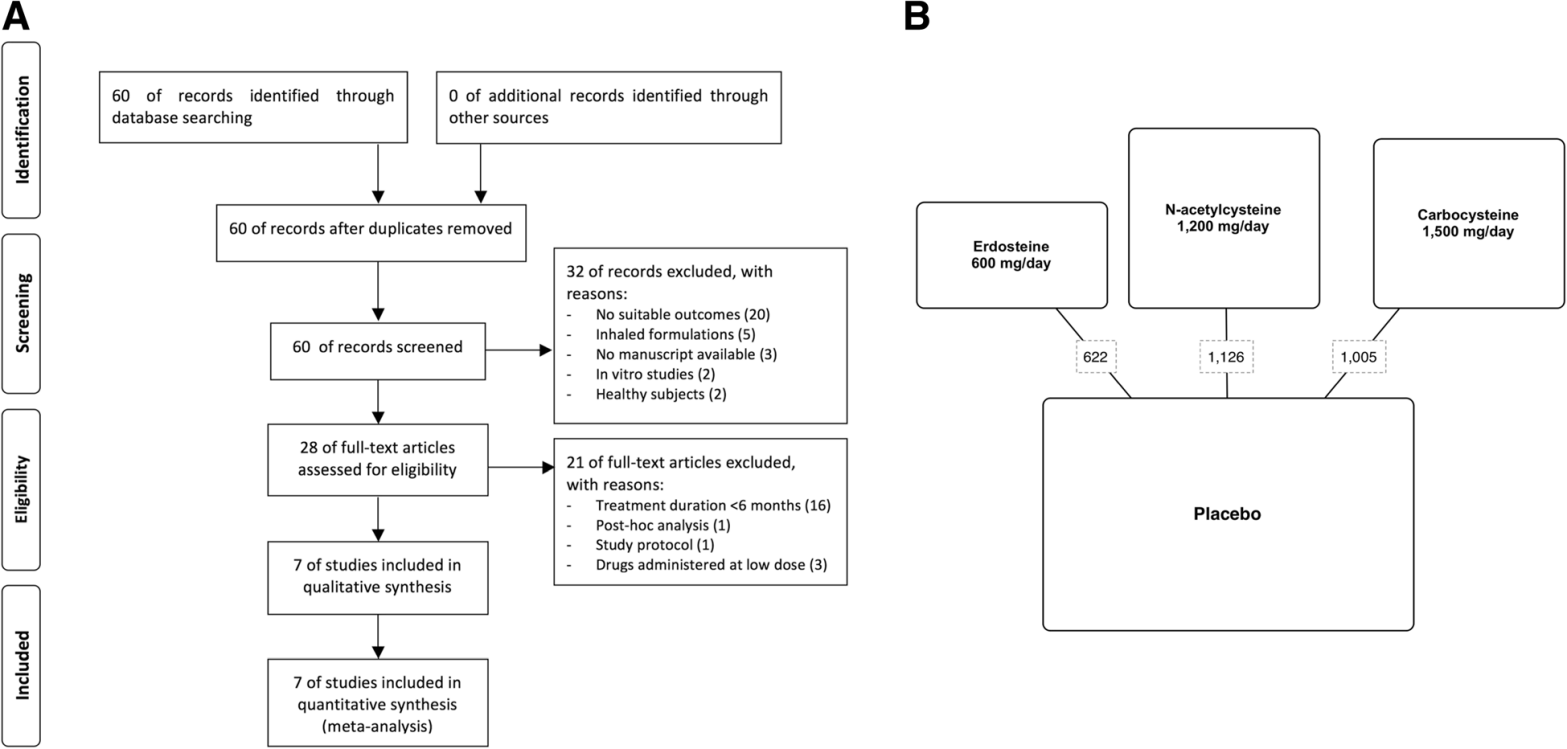
PRISMA flow diagram for the identification of studies included in the meta-analysis (**a**) and diagram displaying the network across the treatments; the links between nodes indicate the direct comparisons between pairs of treatments; the numbers shown along the link lines indicate the number of patients comparing pairs of treatments head-to-head (**b**)

This meta-analysis has been registered in the international database of prospectively registered systematic reviews (PROSPERO registration number: CRD42016053762), and performed in agreement with the PRISMA-P [11], with the flow diagram reported in Fig. 1A. This quantitative synthesis satisfied all the recommended items reported by the PRISMA-P checklist [11].

Two reviewers performed a comprehensive literature search for RCTs evaluating the influence of mucolytic and antioxidant agents in COPD patients. The PICO (Patient problem, Intervention, Comparison, and Outcome) framework was used to develop the literature search strategy, as previously described [12]. Namely, the “Patient problem” included subject affected by COPD; the “Intervention” regarded the administration of mucolytic and antioxidant agents; the “Comparison” was performed with regard to placebo and across each active treatment; the “Outcomes” were the duration of AECOPD, hospitalizations due to AECOPD, and adverse events (AEs).

The terms “erdosteine” OR “carbocysteine” OR “NAC” AND “COPD” AND “clinical trial” were searched in Cochrane Central Register of Controlled Trials (CENTRAL), MEDLINE, Embase, Scopus, Web of Science, ClinicalTrials.gov and EU Clinical Trials Register databases in order to provide for relevant studies available up to November 26, 2018. No language restriction was applied. The following Query Translation was used: “((“erdosteine”[Supplementary Concept] OR “erdosteine”[All Fields]) OR (“carbocysteine”[MeSH Terms] OR “carbocysteine”[All Fields]) OR (“acetylcysteine”[MeSH Terms] OR “acetylcysteine”[All Fields] OR “n acetylcysteine”[All Fields])) AND (“pulmonary disease, chronic obstructive”[MeSH Terms] OR (“pulmonary”[All Fields] AND “disease”[All Fields] AND “chronic”[All Fields] AND “obstructive”[All Fields]) OR “chronic obstructive pulmonary disease”[All Fields] OR “copd”[All Fields]) AND Clinical Trial[ptyp]”.

Citations of previously published meta-analyses and relevant reviews were examined to identify further pertinent studies, if any [3, 5, 13–15].

### Study selection

Published RCTs involving COPD patients treated for more than 6 months with oral formulations of erdosteine 600 mg/day, carbocysteine 1500 mg/day, and NAC 1200 mg/day were included in this meta-analysis.

Two reviewers independently checked the relevant studies identified from literature searches obtained from the already mentioned databases. The studies were selected in agreement with the above-mentioned criteria, and any difference in opinion about eligibility was resolved by general consensus.

### Endpoints

The primary endpoint of this meta-analysis was the impact of erdosteine 600 mg/day, carbocysteine 1500 mg/day, and NAC 1200 mg/day on the reduction in the risk of AECOPD, compared to placebo and across each active treatment.

The secondary endpoints were the impact of erdosteine 600 mg/day, carbocysteine 1500 mg/day, and NAC 1200 mg/day on the risk of experiencing at least on AECOPD, the duration of AECOPD, and the risk of hospitalization due to AECOPD, compared to placebo. The frequency of AEs was another secondary endpoint.

### Quality score, risk of bias and evidence profile

The Jadad score, with a scale of 1 to 5 (score of 5 being the best quality), was used to assess the quality of the RCTs concerning the likelihood of biases related to randomization, double blinding, withdrawals and dropouts [16]. A Jadad score ≥ 3 was defined to identify high quality studies. Two reviewers independently assessed the quality of individual studies, and any difference in opinion about the quality score was resolved by consensus.

In the pairwise meta-analysis moderate to high levels of heterogeneity between-studies were considered for I^2^ > 50%; the risk of publication bias was assessed for primary endpoints by applying the funnel plot and Egger’s test, as previously described [16]. Evidence of asymmetry from Egger’s test was considered to be significant at *P* < 0.1, and the graphical representation of 90% confidence bands have been presented [16]. The risk of bias in the network meta-analysis was checked via the consistency/inconsistency analysis to assess whether the outcomes resulting from the consistency and inconsistency models fit adequately with the line of equality, as previously described [5].

The quality of the evidence was assessed for the primary endpoint in agreement with the Grading of Recommendations Assessment, Development, and Evaluation (GRADE) system, indicating ++++ for high quality of evidence, +++ for moderate quality of evidence, ++ for low quality of evidence, and + for very low quality of evidence [17].

### Data extraction

Data from included RCTs were extracted and checked for study characteristics and duration, enrolled patients, drugs and daily doses, disease characteristics, AECOPD definition, history and rate of AECOPD, age, gender, smoking habit, lung function, and Jadad score. Due to the complexity of this meta-analysis, data have been extracted in agreement with DECiMAL recommendations [18].

### Data analysis

A pairwise meta-analysis was performed to quantify the impact of erdosteine 600 mg/day, carbocysteine 1500 mg/day, and NAC 1200 mg/day on primary and secondary endpoints, compared to placebo.

The follow-up duration could be not consistent across the RCTs included in this meta-analysis. Therefore, the data concerning the risk assessment have been normalized as a function of person-time (namely person-season), where one season lasts three months [19]. This method involves the conversion of the measures into a common metric (events per person-time) prior to meta-analyze the data, leading to increased estimates of effect, precision, and clinical interpretability of results [20, 21]. Specifically, the numerator represents the count of total events and the denominator represents the given time duration multiplied by the number of patients [22]. Such a method has been supported by the Cochrane Collaboration and successfully used in recent meta-analyses [20, 21, 23, 24].

Results of the pairwise meta-analysis are expressed as relative risk (RR) or mean difference (MD), and 95% confidence interval (95%CI). Since data were selected from a series of studies performed by researchers operating independently, and a common effect size cannot be assumed, binary random-effects model was used in order to balance the study weights and adequately estimate the 95%CI of the mean distribution of drugs effect on the investigated variables [16].

A network meta-analysis was also carried out to perform a comparison across the investigated agents with respect to the primary endpoint, and to rank their efficacy in reducing the risk on AECOPD. The network meta-analysis was carried out by including exclusively high quality RCTs that introduced neither heterogeneity nor bias in the overall effect estimate of primary endpoint. Since heterogeneity and bias may propagate through a network of RCTs, and thus affect the estimates differentially across regions of the network, this approach permitted to identify those studies that might alter the correct results of the network meta-analysis [25].

Full Bayesian evidence network was used was used in the network meta-analysis (chains: 4; initial values scaling: 2.5; tuning iterations: 20.000; simulation iterations: 50.000; tuning interval: 10). The convergence diagnostics for consistency and inconsistency was assessed via the Brooks-Gelman-Rubin method, as previously described [26]. Results of the network meta-analysis are expressed as relative effect (RE) and 95% credible interval (95%CrI). The probability that each intervention arm was the most effective was calculated by counting the proportion of iterations of the chain in which each intervention arm had the highest mean difference, and the surface under the cumulative ranking curve (SUCRA), representing the summary of these probabilities, was also calculated. The SUCRA is 1 when a treatment is certain to be the best, and 0 when a treatment is certain to be the worst [27].

Sensitivity analysis was performed to identify the studies that introduced heterogeneity and bias in the effect estimate of primary endpoint.

The analysis of the number needed to treat (NNT) was performed on primary endpoint. NNT is the reciprocal of the absolute risk reduction associated with an intervention over a fixed period of time [28–30]. The values of NNT are reported in this study as person-based and calculated by analyzing the Kaplan-Meier curves or by using the raw data provided in the primary publications, as previously described [31, 32]. The relative weight of each study resulting from the pairwise meta-analysis was used to calculate the weighted average rate of the investigated arms and to correctly provide NNT values.

The safety profile of the investigated treatments was investigated through a pooled analysis of the frequency of AEs, which was ranked in agreement with European Medicine Agency (EMA) guidelines: very common ≥1/10, common ≥1/100 to < 1/10, uncommon ≥1/1000 to < 1/100, frequency not known if not calculable from the extracted data.

OpenMetaAnalyst [33] and GeMTC [34] software were used for performing the meta-analysis, GraphPad Prism (CA, US) software to graph the data, and GRADEpro GDT to assess the quality of evidence [17]. The statistical significance for the effect estimates resulting from the pairwise and network meta-analyses was assessed for *P* < 0.05.

## Results

### Studies characteristics

Results obtained from 2753 COPD patients (11.15% treated with erdosteine, 18.27% treated with carbocysteine, 20.41% treated with NAC, and 50.16% treated with placebo) were selected from 7 RCTs [6–10, 35, 36] published between 2004 and 2017. The relevant characteristics of studies, disease, and patients and the definition of AECOPD are described in Table 1; Fig. 1B shows the network across the treatments.

**Table 1:** Patient demographics, baseline and study characteristics

Study, authors, year, trial registration, and reference	Study characteristics	Study duration (weeks)	Enrolled patients	Drugs and daily doses	Disease characteristics	AECOPD definition	Patient with AECOPD history (%)	AECOPD in the previous year (rate)	Age (years)	Male (%)	Current smokers (%)	Smoking history (pack-years)	Post-bronchodilator FEV1 (% predicted)	Jadad score
RESTORE, Dal Negro et al., 2017, NCT NCT01032304 [6]	Multicentre, double-blind, randomized, placebo-controlled, parallel-group	52	467	Erdosteine, 600 mg	FEV1 ≥ 30% and ≤ 70%	“A symptomatic worsening beyond normal day-to-day variations and requiring a change in regular medication and/or health care resources utilisation (e.g. increased use of bronchodilators, treatment with antibiotics and/or systemic corticosteroids, visit to an emergency department, hospitalization)”	100	2.3	65	74	29	> 10	52	5
PANTHEON, Zheng et al., 2014, ChiCTR-TRC-09000460 [7]	Multicentre, double-blind, randomized, placebo-controlled, parallel groups	52	1006	N-acetylcysteine, 1200 mg	FEV1 ≥ 30% and ≤ 70%	“At least a 2 day persistence of two (type II moderate) or all three (type III, severe) major symptoms (worsening dyspnoea, increase in sputum purulence or volume), or of any one major symptom plus at least one minor symptom (type I, mild) (upper airway infection, unexplained fever, and increased wheezing).”	100	1.8	66	82	18	36	49	4
HIACE, Tse et al., 2013, NCT01136239 [8]	Single-centre, double-blind, randomized placebo-controlled, parallel groups	52	120	N-acetylcysteine, 1200 mg	FEV1: NA	“Two of the following three symptoms: increase in shortness of breath, volume, or purulence of sputum.”	NA	2	71	93	23	NA	60	4
PEACE, Zheng et al., 2008, UMIN-CRT C000000233 [9]	Multicentre, double-blind, randomized, placebo-controlled, parallel groups	52	707	Carbocysteine, 1500 mg	FEV1 ≥ 25% and ≤ 79%	“At least 2-day persistence of at least two major symptoms (worsening dyspnoea and an increase in sputum purulence, volume, or both), or of any single major symptom plus more than one minor symptom (upper airway infection, unexplained fever, and increased wheezing).”	100	NA	65	79	74.5 (ever smokers)	NA	45	5
Tatsumi et al., 2007, NA [35]	Multicenter, randomized, parallel groups	52	142	Carbocysteine, 1500 mg	FEV1 < 80%	“Changes in the following symptoms from their stable condition according to the Anthonisen criteria: dyspnea, sputum purulence, sputum volume, cold, wheeze, cough, fever, and change in respiratory rate or heart rate of 20%.”	100	NA	70	92	NA	NA	< 70	1
Yasuda et al., 2006, NA [36]	Randomized, double blind, placebo-controlled, parallel groups	52	156	Carbocysteine, 1500 mg	FEV1 ≥ 30%	“An acute and sustained worsening of COPD symptoms requiring changes to regular treatment, as previously described.”	NA	NA	73	85	NA	44	62	3
Moretti et al., 2004, NA [10]	Multicentre, randomized, double-blind, placebo-controlled, parallel groups	32	155	Erdosteine, 600 mg	FEV1 < 70%	“New episodes of acute disease with muco-purulent or purulent sputum, cough and at least two of the following symptoms: general malaise, fever > 38 °C, breathlessness, difficulty in expectoration and leukocytosis.”	100	NA	68	80	33	> 20	59	3

*AECOPD* acute exacerbation of COPD, *COPD* chronic obstructive pulmonary disease, FEV_1_: forced expiratory volume in 1 s; NA: not available

Five RCTs were published as full-text papers [6–10], and two RCTs as letters to the editor [35, 36]. Six RCTs were published as high quality studies (Jadad score ≥ 3) [6–10, 36], and one as low-quality study (Jadad score = 1) [35]. The duration of treatment ranged from 32 weeks to 52 weeks.

#### Primary endpoint

**Fig. 2:**
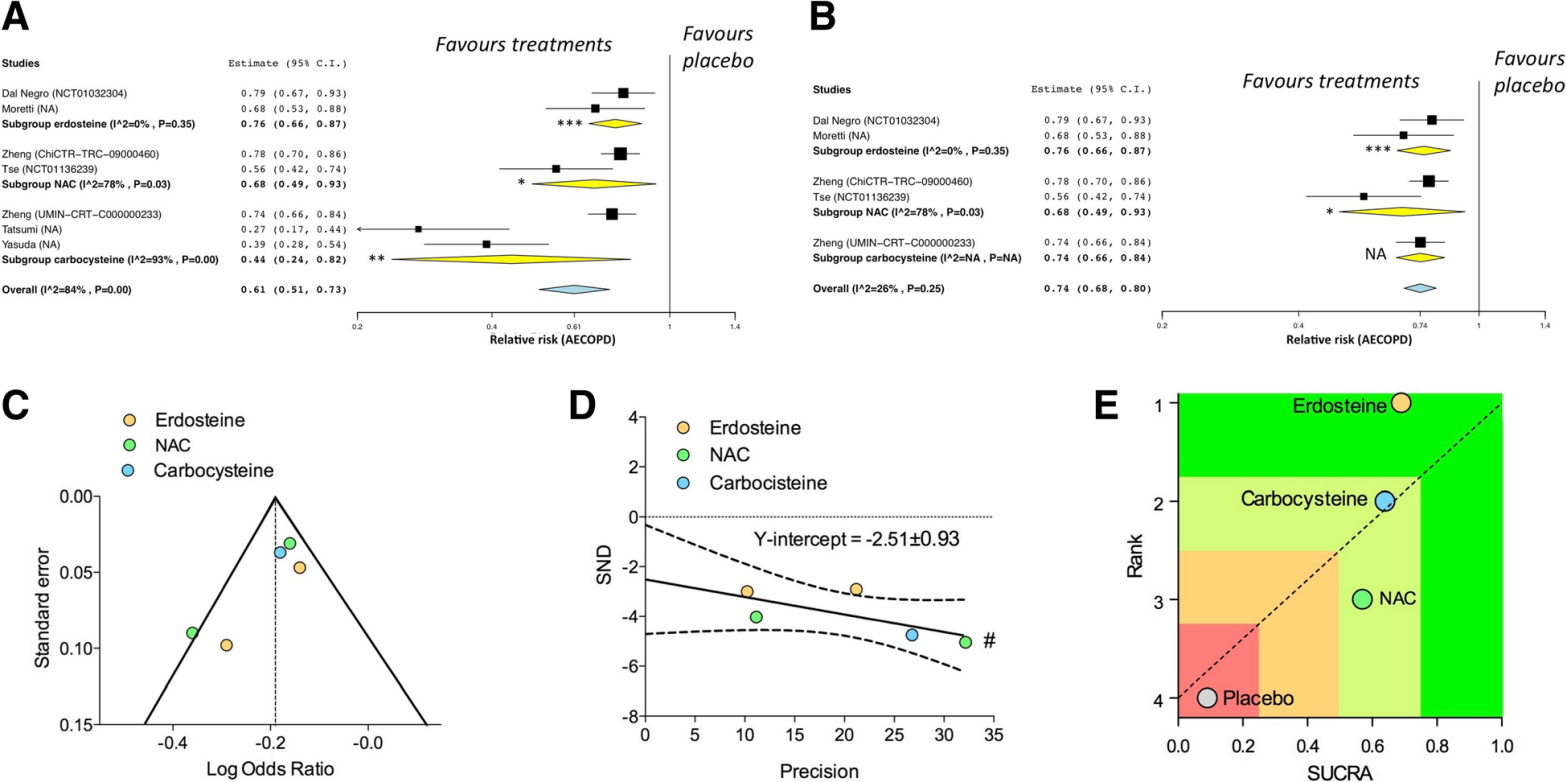
Forest plot of pair-wise meta-analysis of primary endpoints: impact of the erdosteine, carbocysteine, and NAC on the risk of AECOPD vs. placebo (**a**); sensitivity analysis performed by excluding the studies that introduced significant heterogeneity in the overall effect estimate (**b**); publication bias assessment via funnel plot (**c**) and Egger’s test (**d**); ranking plot resulting from the network meta-analysis in which treatments were plotted on X-axis according to SUCRA (score of 1 being the most effective) and on Y-axis according to the rank of being the best treatment (score of 1 being the most effective) (**e**). ^#^*P* < 0.1, *P < 0.05, **P < 0.01, and ***P < 0.001. *AECOPD* acute exacerbation of COPD, *COPD* chronic obstructive pulmonary disease, *NA* not available, *NAC* N-acetylcysteine, *SND* standard normal deviate, *SUCRA* surface under the cumulative ranking curve

The pairwise meta-analysis indicated that erdosteine, carbocysteine, and NAC both significantly reduced the risk of AECOPD. The overall effect estimate was affected by high and significant heterogeneity that was driven by the studies on carbocysteine and NAC. Conversely, the effect estimate resulting for erdosteine was free from any heterogeneity (Fig. 2A).

The sensitivity analysis indicated that the main source of heterogeneity was introduced by the studies of Tatsumi et al. and Yasuda et al. on carbocysteine [35, 36], as excluding these RCTs the overall heterogeneity reduced at acceptable and not significant levels (I^2^ 26%, *P* > 0.05) (Fig. 2B). Nevertheless, the visual inspection of funnel plot indicated that the study of Tse et al. [8] introduced a certain level of publication bias (Fig. 2C). In fact Egger’s test indicated that the results of sensitivity analysis were affected by significant publication bias, as the regression line and 90% confidence bands did not intercept the origin of the graph (Fig. 2D). A further sensitivity analysis confirmed that the study of Tse et al. [8] was a source of publication bias, as the regression line and 90% confidence bands of Egger’s test intercepted the origin of the graph (Y-intercept − 1.64, − 6.23 to 3.00) when this RCT [8] was removed from the analysis.

The results of the network meta-analysis showed that there was not significant difference (*P* > 0.05) across the investigated drugs with respect to their effect against the risk of AECOPD (erdosteine vs. carbocysteine: RE 0.97, 95%CrI 0.34–2.56; carbocysteine vs. NAC: RE 0.95, 95%CrI 0.29–2.92; erdosteine vs. NAC: RE 0.92, 95%CrI 0.32–2.38). However, the network meta-analysis indicated that erdosteine was the most effective agent in preventing the risk of AECOPD (upper quartile in the SUCRA ranking), followed by carbocysteine and NAC (third quartile in the SUCRA ranking) (Fig. 2E).

The consistency/inconsistency analysis showed that all points fit adequately with the line of equality (goodness of fit: R^2^ 0.99; slope 0.96, 95%CI 0.90–1.02), indicating that the network meta-analysis was not affected by significant bias.

The person-based NNT analysis reported that 10.11 (95%CI 5.41–76.39) patients had to be treated with erdoisteine for one year to prevent one AECOPD, compared to placebo (*P* < 0.05). Conversely, the NNT values for both carbocysteine (30.92, 95%CI 8.61 - ∞) and NAC (15.69, 95%CI 7.31 - ∞) were not significantly different (*P* > 0.05) than placebo.

The GRADE analysis indicated high quality of evidence (++++) for erdosteine, low quality of evidence (++) for carbocysteine, and moderate quality of evidence (+++) for NAC, when compared to placebo. The quality of evidence in the network meta-analysis comparing erdosteine, carbocysteine, and NAC via placebo arm was moderate (+++).

#### Secondary endpoints

**Fig. 3:**
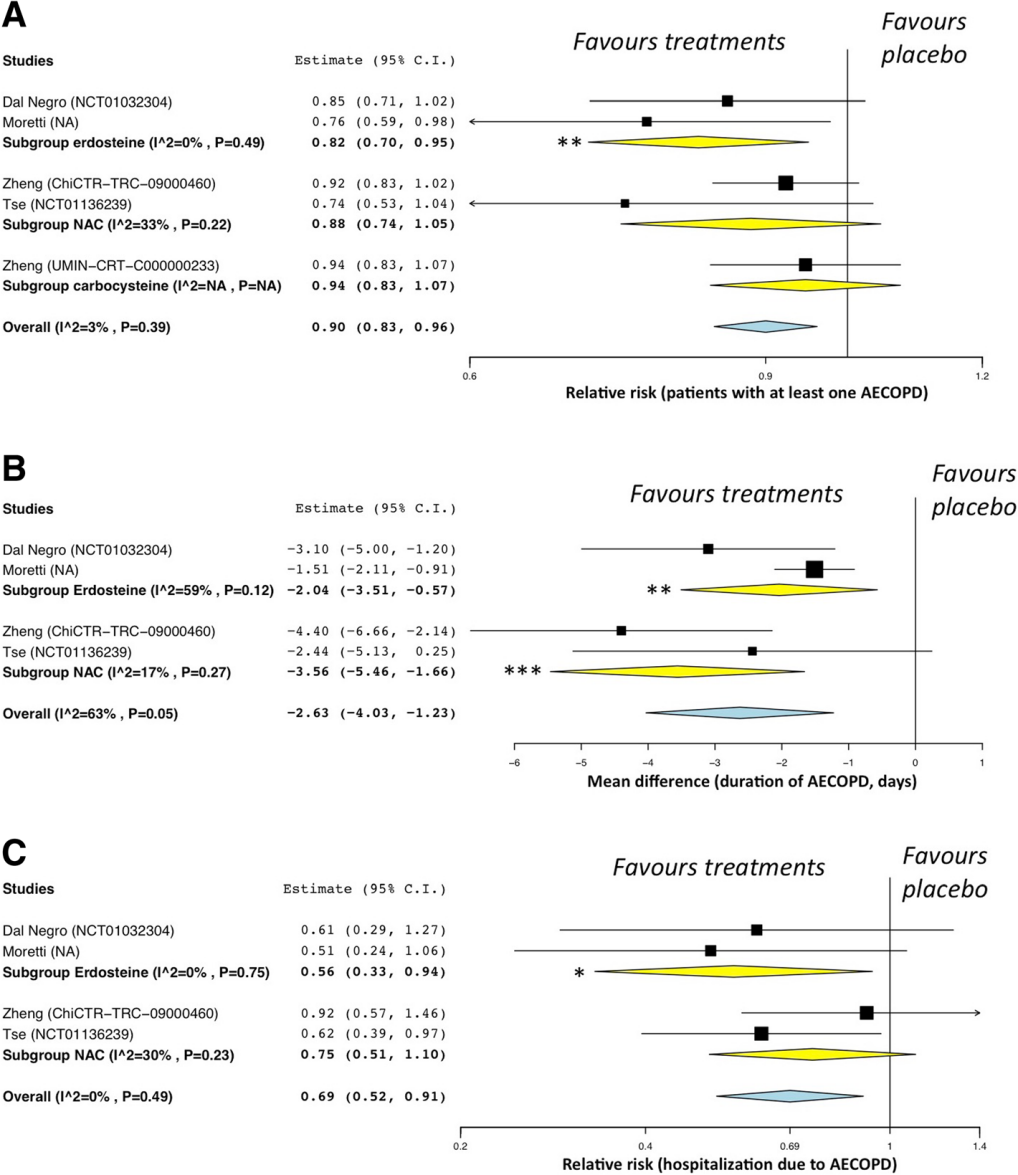
Forest plot of pair-wise meta-analysis of secondary endpoints: impact of the erdosteine, carbocysteine, and NAC on the risk of experiencing at least one AECOPD (**a**), duration of AECOPD (**b**), and risk of hospitalization due to AECOPD (**c**), vs. placebo. *P < 0.05 and **P < 0.01. AECOPD: acute exacerbation of COPD; COPD: chronic obstructive pulmonary disease

Erdosteine, but neither carbocysteine nor NAC, significantly (*P* < 0.01) reduce the risk of experiencing at least one AECOPD, compared to placebo (Fig. 3A). Erdosteine and NAC both significantly (P < 0.01 and *P* < 0.001, respectively) reduced the duration of AECOPD, compared to placebo; no data on this outcome are currently available for carbocysteine (Fig. 3B). Erdosteine, but not NAC, significantly (*P* < 0.05) reduced the risk of hospitalization due to AECOPD, compared to placebo; no data on this outcome are currently available for carbocysteine (Fig. 3C).

The pooled analysis of safety profile showed that AEs were very common in patients treated with NAC, and common in those treated with either erdosteine and carbocysteine, or that received placebo. However, AEs were mild in severity and generally well tolerated. The most frequent AEs was respiratory tract infection (NAC: 10.85%, carbocysteine: 0.56%, erdosteine: not detectable), and detailed frequencies of further specific AEs are reported in Table 2.

**Table 2:** Pooled analysis of AEs extracted from the studies on erdosteine, carbocysteine, and NAC in COPD patients and ranked by frequency in agreement with EMA guidelines [37]

	Erdosteine	Carbocysteine	NAC	Placebo
Total number of subjects	354	557	553	1151
Frequency (%) of all AEs	1.32 (+++)	2.26 (+++)	18.26 (++++)	8.43 (+++)
Frequency (%) of specific AEs:				
respiratory tract infection	ND	0.56 (+++)	10.85 (++++)	4.26 (+++)
gastrointestinal disorders	0.44 (++)	1.13 (+++)	4.16 (+++)	4.26 (+++)
pruritus	ND	ND	1.08 (+++)	2.69 (+++)
cerebrovascular disorders	0.44 (++)	ND	0.90 (++)	0.09 (+)
dizziness	ND	ND	0.72 (++)	0.09 (+)
musculoskeletal disorders	ND	0.28 (++)	0.54 (++)	0.78 (++)
hepatobiliary disorders	0.44 (++)	ND	ND	ND
malaise	ND	0.28 (++)	ND	0.09 (+)
insomnia	ND	ND	ND	0.26 (++)
increased cough	ND	ND	ND	0.17 (++)

++++: very common (≥1/10); +++: common (≥1/100 to < 1/10); ++: uncommon (≥1/1000 to < 1/100); +: rare (≥1/10,000 to < 1/1000); AEs: adverse events; COPD: chronic obstructive pulmonary disease; EMA: European Medicine Agency; NAC: N-acetylcysteine; ND: not detectable (frequency not known)

## Discussion

In this quantitative synthesis of current literature both erdosteine, carbocysteine, and NAC fulfilled the primary endpoint represented by the reduction in the risk of AECOPD. Considering exclusively the high-quality RCTs that did not introduce significant heterogeneity in the pairwise meta-analysis, this study indicates that the mean effect estimate of the overall impact of mucolytic/antioxidant agents reached the minimal clinically important difference (MCID: ≈0.75 RR) [19, 38] in reducing the risk of AECOPD compared to placebo. In any case, the results of pairwise meta-analysis seem to be affected by a certain level of publication bias that was mainly related with the results extracted from the study of Tse et al. [8], as confirmed by both funnel plot and Egger’s test analyses.

Although we found no significant difference across the investigated drugs with respect to their protective effect against AECOPD, the SUCRA analysis resulting from the network meta-analysis indicated that erdosteine was the most effective agent, followed by carbocysteine and NAC. Interestingly, the consistency/inconsistency analysis showed that the network meta-analysis was not affected by significant bias.

The superiority of erdosteine with respect to carbocysteine and NAC was also confirmed by the NNT analysis, that provided significant NNT values for erdosteine but neither for carbocysteine nor for NAC, when compared to placebo. Specifically, ≈10 patients had to be treated with erdoisteine for one year to prevent one AECOPD.

The analysis of the quality of evidence concerning the efficacy on risk of AECOPD compared to placebo indicates that we can be very confident that the true effect of erdosteine lies close to that of the estimate of the effect. Conversely, moderate to limited confidence resulted for NAC and carbocysteine, respectively. This means that while further research is very unlikely to change our confidence in the estimate of effect resulting for erdosteine, future studies are likely to very likely to have an important impact on the confidence in the estimate of effect resulting for NAC and carbocysteine [39]. Due to the indirect comparison across erdosteine, carbocysteine, and NAC in the Bayesian process, the quality of evidence of network meta-analysis is moderate, thus representing the main limitation of our study.

Further limits of this quantitative synthesis are represented by the difference, or missing data, concerning the baseline characteristics of COPD patients enrolled in the RCTs and included in the meta-analysis, namely the current smoking levels, respiratory function impairment, and rate of AECOPD in the previous year. Besides, also the definition of AECOPD was not consistent across the studies included in this meta-analysis. The risk assessment was normalized as a function of person-time since the follow-up was not consistent across the RCTs included in the meta-analysis. Although this procedure has been extensively validated and supported by the Cochrane Collaboration [20, 21], adjusting data for different follow-up duration may represent another minor limitation of the study.

Concerning the secondary endpoints, only erdosteine was significantly effective in reducing the risk of experiencing at least one AECOPD and the risk of hospitalization due to AECOPD, whereas the duration of AECOPD was significantly reduced by both erdosteine and NAC.

The pooled analysis of AEs indicates that the mucolytic/antioxidant agents investigated in this study are characterized by a positive safety profile, and that the recorded AEs were mild in severity and generally well tolerated.

Indeed the efficacy and safety profile of mucolytic/antioxidant agents resulting by quantitative synthesis of the current literature supports the use of erdosteine, carbocysteine, and NAC in COPD patients, as recommended by the last (GOLD) document [1]. Considering that most the pivotal RCTs [6, 7, 9] explored the impact of mucolytic/antioxidant agents on the rate of overall AECOPD, that includes both mild, moderate and severe exacerbations, to date it is still unclear whether these drugs can be effective in specifically reducing the risk of moderate or severe AECOPD. Furthermore, considering the moderate quality of evidence resulting from the network meta-analysis, and the lack of significant difference across the investigated drugs, we cannot exclude that future study may change the rank provided by the SUCRA analysis.

## Conclusion

Concluding, the current evidence suggests that the overall efficacy/safety profile of erdosteine is superior to that of both carbocysteine and NAC. However, future head-to-head studies performed on the same COPD populations are needed to definitely confirm the results of this quantitative synthesis.

## Data Availability

The datasets used and/or analysed during the current study are available from the corresponding author on reasonable request.

## Abbreviations

AECOPD: acute exacerbation of COPD
CENTRAL: Cochrane Central Register of Controlled Trials
CI: confidence interval
COPD: chronic obstructive pulmonary disease
CrI: credible interval
EMA: European Medicine Agency
GOLD: Global Initiative for Chronic Obstructive Lung Disease
GRADE: Grading of Recommendations Assessment, Development, and Evaluation
ICS: inhaled corticosteroid
MCID: minimal clinically important difference
MD: mean difference
NAC: N-acetylcysteine
NNT: number needed to treat
PANTHEON: Placebo-controlled study on efficAcy and safety of N-acetylcysTeine High dose in Exacerbations of chronic Obstructive pulmoNary disease
PICO: Patient problem, Intervention, Comparison, and Outcome
PRISMA: Preferred Reporting Items for Systematic Reviews and Meta-Analyses
RCT: randomized controlled trial
RE: relative effect
RESTORE: Reducing Exacerbations and Symptoms by Treatment with ORal Erdosteine
RR: relative risk
SUCRA: surface under the cumulative ranking curve
